# Fabrication and Characterization of *Ampelopsis grossedentata* Leaf Powder-Stabilized Pickering Emulsion Gels

**DOI:** 10.3390/foods15173086

**Published:** 2026-08-31

**Authors:** Si Chen, Yanyu Li, Benguo Liu

**Affiliations:** School of Food Science, Henan Institute of Science and Technology, Xinxiang 453003, China; chensi@hist.edu.cn (S.C.); 2484365823@stu.hist.edu.cn (Y.L.)

**Keywords:** *Ampelopsis grossedentata*, Pickering emulsion gels, plant-based particulate stabilizers, lutein delivery, interfacial stability

## Abstract

Natural carrier systems that integrate structural integrity with protective function are critical for the efficient delivery of lipophilic bioactives. Here, we employed *Ampelopsis grossedentata* leaf powder (AGP), a flavonoid-rich plant material, as a particulate stabilizer to construct Pickering emulsion gels. The influence of AGP concentration and oil phase volume fraction on gel structure was examined, and the ability of the gels to protect lutein was further assessed. AGP displayed appropriate interfacial wettability, enabling it to adsorb at the oil–water interface and build a coherent particle layer. A progressive increase in AGP concentration reduced droplet size and raised gel strength from 3.07 × 10^−2^ N to 5.04 × 10^−2^ N, reflecting the formation of a more robust particle-based network. Among the formulations tested, the gel prepared with 4% AGP gave the best overall structural performance: it had the highest gel strength, together with favorable elasticity index (EI) and macroscopic viscosity index (MVI) values, indicative of superior load-bearing capacity and disturbance resistance. Raising the oil phase fraction further tightened droplet packing and improved structural stability. In storage trials, the optimized AGP-stabilized emulsion gel retained substantially more lutein than the MCT oil system without powder, with the 7-day retention increasing from 14.9% to 55.9%. This protective effect may be attributed to the physical barrier created by interfacial particles, together with the antioxidant activity of flavonoids naturally present in AGP. These findings uncover a synergistic role of plant-based particles in structuring Pickering emulsion gels and preserving lipophilic bioactives, highlighting AGP as a sustainable and functional platform for delivery systems.

## 1. Introduction

Lipophilic bioactive compounds possess considerable potential in functional food applications due to their nutritional and physiological benefits. However, their limited water dispersibility and susceptibility to degradation induced by oxygen, light, and heat greatly restrict their practical utilization. Emulsion-based delivery systems have been widely explored to improve the aqueous dispersion of hydrophobic compounds and enhance their storage stability [1]. Nevertheless, conventional emulsions generally rely on surfactants or proteins to maintain interfacial integrity, and the resulting interfacial layers may still be vulnerable during storage, leading to droplet coalescence and the loss of encapsulated bioactive compounds [2]. Therefore, the development of emulsion systems with reinforced interfacial strength and improved structural stability remains an important research topic in food colloid science.

Pickering emulsions are stabilized by the irreversible adsorption of solid particles at the oil–water interface, where the particle layer provides enhanced mechanical protection compared with conventional molecular emulsifiers. By further promoting interparticle interactions and constructing a continuous three-dimensional network, Pickering emulsions can be transformed into gel-like structured systems, resulting in improved resistance to phase separation and tunable rheological properties [3]. In recent years, the stabilizing particles used for Pickering emulsions have expanded from conventional inorganic materials to natural components, including proteins, polysaccharides, and plant-derived particles [4]. Compared with purified single-component materials, plant powders retain inherent tissue structures and diverse bioactive constituents. Their abundant proteins, polysaccharides, dietary fibers, and phenolic compounds can collectively regulate particle surface characteristics, interfacial adsorption behavior, and the formation of continuous-phase networks, providing new opportunities for developing Pickering systems with both structural functionality and bioactivity [5].

*Ampelopsis grossedentata leaves* (Tengcha tea) are a flavonoid-rich plant resource, in which dihydromyricetin (DMY) and other phenolic compounds represent the major bioactive constituents and contribute to its antioxidant capacity [6]. Previous studies have demonstrated that DMY possesses strong Pickering emulsifying activity [7]. However, the production of purified DMY generally involves multiple processing steps, including solvent extraction, separation, and purification, which increases processing complexity and may limit its broader application in food colloidal systems. Compared with isolated compounds, *Ampelopsis grossedentata* leaf powder (AGP) retains the inherent plant microstructure and diverse natural components, including polysaccharides, proteins, dietary fibers, and phenolic compounds. These components may collectively regulate particle surface characteristics, wettability, and interfacial affinity, thereby potentially facilitating particle adsorption at the oil–water interface and contributing to the formation of continuous-phase particle networks [8]. Therefore, this study employed AGP as a solid particle stabilizer and medium-chain triglyceride (MCT) as the oil phase to fabricate Pickering emulsion gels. The effects of AGP concentration and oil phase fraction on structural formation were investigated through comprehensive analyses of particle characteristics, interfacial behavior, microstructure, gel strength, and microrheological properties. Furthermore, the protective effect of the developed emulsion gels on lutein stability during storage was evaluated.

## 2. Materials and Methods

### 2.1. Materials and Chemicals

AGP (moisture content, 7.70 ± 0.43%) was purchased from Enshi Zhiyi Tea Co., Ltd. (Enshi, China). Medium-chain triglyceride (MCT) and lutein standard (purity ≥ 98%) were obtained from Shanghai Yuanye Bio-Technology Co., Ltd. (Shanghai, China). Other chemicals were of analytical grade and used without further purification. Deionized water was used throughout the experiments.

### 2.2. Morphology and Particle Size Distribution of AGP

The morphology of AGP particles was examined using a scanning electron microscope (SEM, Quattra S, FEI, Hillsboro, OR, USA). The powder samples were evenly dispersed onto conductive adhesive tape, followed by vacuum sputter coating with gold. Images were subsequently collected at an accelerating voltage of 5.0 kV.

The particle size distribution of AGP was determined using a laser particle size analyzer (BT-9300H, Better Instrument Co., Ltd., Dandong, China). Prior to measurement, the samples were ultrasonically dispersed in deionized water, and the obscuration value was adjusted to 15–16%. Particle size parameters were automatically calculated using the instrument-associated software, with the refractive index of water set at 1.33.

### 2.3. Interfacial Tension Measurement of AGP

The effect of AGP dispersion on the interfacial tension at the MCT/water interface was evaluated using an optical contact angle analyzer (Theta Lite, Biolin Scientific, Stockholm, Sweden). AGP was dispersed in ultrapure water and sonicated for 10 min at room temperature to facilitate sufficient hydration. The dispersion was then centrifuged at 8500 rpm for 15 min, and the obtained supernatant was collected for interfacial tension measurement. During the measurement, MCT droplets were generated using the pendant drop method. The droplet profiles were fitted according to the Young–Laplace equation, and the corresponding oil–water interfacial tension values were calculated. Ultrapure water without AGP was used as the control.

### 2.4. Contact Angle Measurement of AGP

The wettability of AGP particles in the oil–water system was evaluated using the static contact angle method. AGP was compressed into cylindrical pellets with an approximate diameter of 20 mm and a height of 3 mm, which were subsequently placed in a quartz cell filled with MCT. A 12 μL droplet of ultrapure water was deposited onto the pellet surface using a microsyringe, and the evolution of droplet morphology was recorded continuously for 20 s. The contact angle was calculated by fitting the droplet profile using the Young–Laplace model through OneAttension software (version 3.0, Biolin Scientific, Espoo, Finland).

### 2.5. Preparation of Pickering Emulsion Gels

Following the method reported previously [9], Pickering emulsion gels were fabricated using AGP as a solid particle stabilizer. To investigate the effects of AGP concentration and oil phase proportion on emulsion gel formation, the mass fraction of AGP (w) was varied from 2.0% to 4.0%, while the oil phase volume fraction (φ) was varied from 40% to 80%. A predetermined amount of MCT was added to the AGP aqueous dispersion, and the mixture was homogenized at 15,000 rpm for 3 min using a high-speed homogenizer (Ultra-Turrax T18, IKA, Staufen, Germany) at room temperature. The resulting Pickering emulsion gels were stored at room temperature prior to further analysis.

### 2.6. Particle Size Measurement of Emulsion Gels

The droplet size distribution of the emulsion gels was determined using a laser particle size analyzer. Before measurement, the samples were appropriately diluted with deionized water and transferred into the sample cell. The obscuration value was adjusted to 15–16%, followed by particle size analysis. The droplet size distribution curves and characteristic parameters (D_10_, D_50_, and D_90_) were automatically obtained using the instrument software.

### 2.7. Microstructural Observation of Emulsion Gels

The microstructure of the emulsion gels was observed using an optical microscope (BH200P, Boshida, Shenzhen, China) to evaluate droplet morphology and dispersion characteristics. Fresh samples (20 μL) were placed onto glass slides, covered with coverslips, and immediately examined. Representative microscopic images were captured for further analysis.

### 2.8. Gel Strength Measurement of Emulsion Gels

The emulsion gel samples were transferred into cylindrical containers (20 mL) with a height of 15 mm. Gel strength was measured using a texture analyzer (TA.XT plus, Stable Micro Systems Ltd., Surrey, UK) equipped with a P0.5 probe under the GMIA Gelation mode. The test conditions were set as follows: probe speed of 1.0 mm/s, trigger force of 3.0 g, and penetration distance of 4 mm. The obtained data were collected and analyzed using Exponent 6.1 software.

### 2.9. Microrheological Characterization of Emulsion Gels

The microrheological measurements were performed according to the method reported by Song et al. with slight modifications [8]. A Rheolaser Master optical microrheometer (Formulaction, Toulouse, France) was used to characterize the microstructural evolution and short-term stability of the emulsion gels. Briefly, 20 mL of each sample was transferred into a specialized measurement vial and placed in the testing chamber. Measurements were performed in passive mode by monitoring the Brownian motion of endogenous scattering particles without applying external deformation. The particle motion was continuously recorded using a CCD detector at 25 °C for 15,000 s. The elasticity index (EI) and macroscopic viscosity index (MVI) were automatically calculated using Rheosoft Master 1.4.0.0 software. During analysis, the samples were measured under static conditions without external deformation or disturbance.

### 2.10. Evaluation of the Protective Effect of Emulsion Gels on Lutein

The photostability of lutein in Pickering emulsion gels was evaluated using an accelerated UV-induced oxidation test. According to the method described by Wang et al. [10], lutein was first dissolved in MCT at a concentration of 2.0 mg/mL, and the resulting lutein-loaded MCT was used as the oil phase to prepare Pickering emulsion gels following the procedure described in Section 2.6. The samples were subsequently transferred into 10 mL screw-cap glass vials and exposed to UV irradiation at a distance of 15 cm from a 6 W UV lamp under 30 °C. At predetermined intervals (every 2 days), approximately 100 mg of sample was collected and extracted with 10 mL of ethanol/*n*-hexane mixture (1:1, *v*/*v*). The mixture was vortexed three times (10 s each), followed by centrifugation at 8000 rpm for 10 min. The collected supernatant was appropriately diluted before analysis. The absorbance was measured at 446 nm using a UV–visible spectrophotometer, and lutein content was calculated based on a previously established calibration curve. An MCT system containing the same concentration of lutein but without particulate stabilizers was used as the control. The lutein retention rate was calculated according to Equation (1):

(1) $$\mathrm{Lutein}\;\mathrm{retention}\;=\frac{C_{2}}{C_{1}}\times \;100\%$$

where *C*_1_ represents the initial lutein content in the sample, and *C*_2_ represents the lutein content remaining in the sample after exposure to UV irradiation for a specified period.

### 2.11. Statistical Analysis

Data were obtained from three independent experimental replicates and are reported as mean ± standard deviation (SD). Statistical comparisons were performed with IBM SPSS Statistics 26.0. Group differences were assessed by one-way analysis of variance (ANOVA), and post hoc comparisons were made using Duncan’s multiple range test, with significance accepted at *p* < 0.05. Data processing and figure preparation were completed in OriginPro 2024.

## 3. Results and Discussion

### 3.1. Morphological Characteristics and Particle Size Distribution of AGP

**Figure 1:**
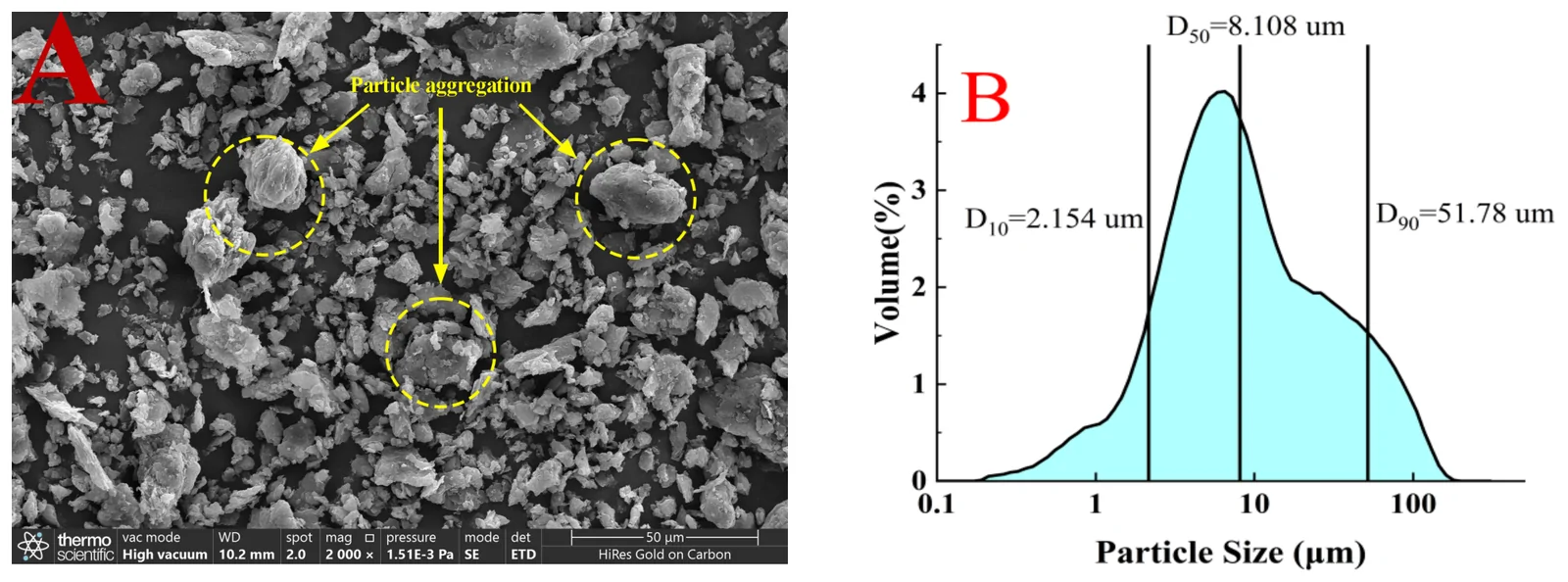
SEM image (**A**) and particle size distribution (**B**) of AGP.

Particle morphology and size distribution are closely related to interfacial adsorption behavior and the stabilization efficiency of Pickering emulsions. In particular, particle size, surface roughness, and morphological features determine their arrangement at the oil–water interface and their ability to form protective interfacial layers [11,12]. As shown in Figure 1A, AGP particles exhibited irregular flake-like and block-like structures with pronounced surface folds and rough textures. Some particle aggregation was also observed. The rough surface morphology may increase the effective contact area between particles and the oil–water interface, potentially favoring interfacial anchoring and promoting the formation of a compact particle layer, thereby improving the stability of Pickering emulsions [13]. The observed particle aggregation also suggests a certain degree of interparticle association, which may facilitate particle packing and network formation in the continuous phase.

The particle size distribution of AGP is presented in Figure 1B. The D_10_, D_50_, and D_90_ values were 2.154, 8.108, and 51.78 μm, respectively, indicating a relatively broad particle size distribution. The D_50_ value of 8.108 μm indicates that AGP mainly consisted of micron-sized particles. The broad particle size distribution may favor multi-scale particle packing, with smaller particles potentially occupying the interstitial spaces between larger particles and contributing to a more compact particle network in the continuous phase [14]. However, these particle size characteristics alone do not provide direct evidence of particle adsorption at the oil–water interface or interfacial coverage, which should be evaluated based on interfacial properties such as wettability and interfacial tension. Taken together, the irregular and rough morphology, particle aggregation, and multiscale particle size characteristics of AGP may favor particle packing and network formation, providing favorable structural conditions for the stabilization of Pickering emulsion gels.

### 3.2. Interfacial Tension of AGP

**Figure 2:**
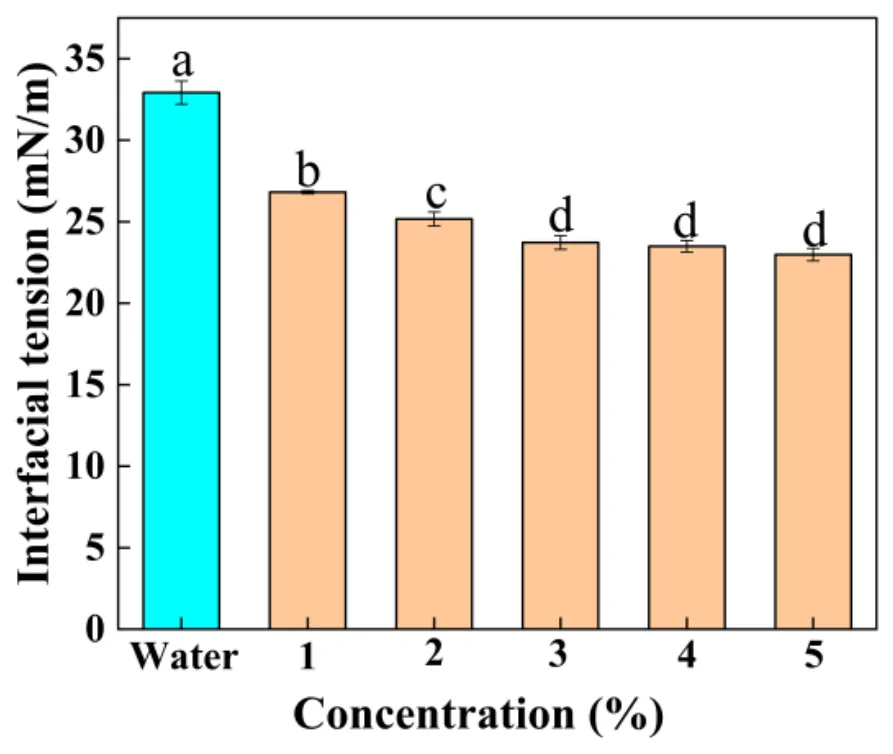
Effect of AGP concentration on interfacial tension. Different lowercase letters indicate significant differences among samples (*p* < 0.05).

Interfacial tension is an important parameter for evaluating the adsorption ability of particles at the oil–water interface and their capacity to reduce interfacial free energy, and it is commonly used to assess the potential of natural particles as Pickering stabilizers. Generally, a lower interfacial tension reflects stronger interfacial activity, facilitating the formation of a compact particle layer at the oil–water interface [15]. As shown in Figure 2, the incorporation of AGP significantly decreased the interfacial tension compared with the pure water system (*p* < 0.05). When the AGP concentration increased from 0 to 1%, the interfacial tension decreased rapidly from 32.92 to 26.81 mN/m [16]. A further increase in AGP concentration to 2% resulted in a slight reduction to approximately 25.17 mN/m. However, when the concentration reached 3% or higher, the interfacial tension remained nearly unchanged, with no significant differences observed among these groups (*p* > 0.05). The reduction in interfacial tension suggests that soluble hydrophilic components and surface-active compounds released from AGP may participate in interfacial regulation by decreasing the free energy of the oil–water interface and promoting particle adsorption [17]. The subsequent plateau at higher AGP concentrations may be associated with the limited available interfacial area for particle occupation. Once the interface becomes saturated, additional particles are mainly retained in the continuous phase, contributing little to further interfacial tension reduction. Similar concentration-dependent behavior has also been observed in Pickering emulsions stabilized by plant-derived particles [18]. The interfacial activity of AGP dispersions may be related to their complex composition, including polysaccharides, proteins, and phenolic compounds. Hydrophilic components facilitate particle dispersion in the aqueous phase, whereas relatively hydrophobic moieties enhance interactions with the oil phase, resulting in amphiphilic characteristics of AGP particles. This balanced wettability promotes particle migration toward the oil–water interface and the formation of a protective particle layer, thereby reducing droplet coalescence and providing an interfacial basis for AGP-stabilized Pickering emulsions [19,20].

### 3.3. Wettability of AGP

**Figure 3:**
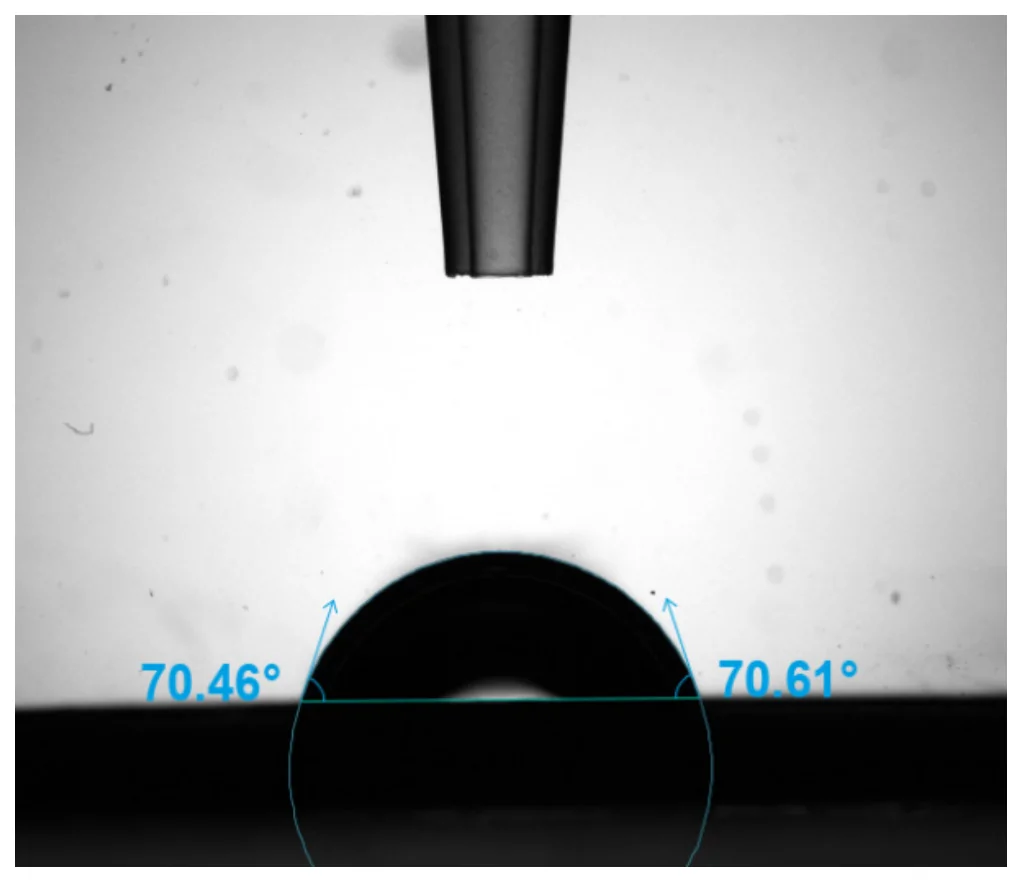
Contact angle of AGP. The arrows indicate the tangent lines used for contact angle measurement.

The contact angle provides direct information on the surface wettability of particles and is widely used to evaluate their interfacial affinity and suitability as Pickering stabilizers. The location of particles at the oil–water interface is closely associated with their hydrophilic–hydrophobic balance. Particles with appropriate wettability can be irreversibly adsorbed at the interface and form a mechanically robust protective layer, thereby improving emulsion stability [21]. As presented in Figure 3, the contact angle of AGP particles was 70.46°, demonstrating a moderate balance between hydrophilicity and hydrophobicity. The contact angle of AGP was approximately 70°, indicating intermediate wettability that is generally favorable for particle positioning and anchoring at the oil–water interface, which is consistent with the Pickering stabilization theory [22]. In contrast, excessively high or low contact angles may cause particles to predominantly reside in the oil or aqueous phase, respectively, reducing their interfacial stabilization efficiency [13,23]. Therefore, the contact angle of AGP (~70°) provides an appropriate wetting balance for effective adsorption at the oil–water interface and the formation of a continuous particle-based interfacial layer. The favorable wettability of AGP may originate from its complex surface composition. Plant-derived particles generally contain various components, which collectively determine surface polarity and hydrophobic characteristics. Hydrophilic groups in polysaccharides and proteins enhance interactions with the aqueous phase, whereas phenolic compounds, lipids, and other hydrophobic compounds contribute to oil affinity, facilitating particle attachment at the oil–water interface [24]. Moreover, combined with the SEM observations described above, the irregular morphology and rough surface structure of AGP particles may promote contact-line pinning at the interface, thereby increasing the resistance to particle detachment [25]. These characteristics are beneficial for establishing a stable Pickering emulsion. Overall, the moderate wettability of AGP particles provides favorable interfacial activity and stabilization capability, laying a structural foundation for the subsequent fabrication of Pickering emulsion gels and the improvement of nutraceutical delivery performance.

### 3.4. Macroscopic Appearance of Pickering Emulsion Gels

**Figure 4:**
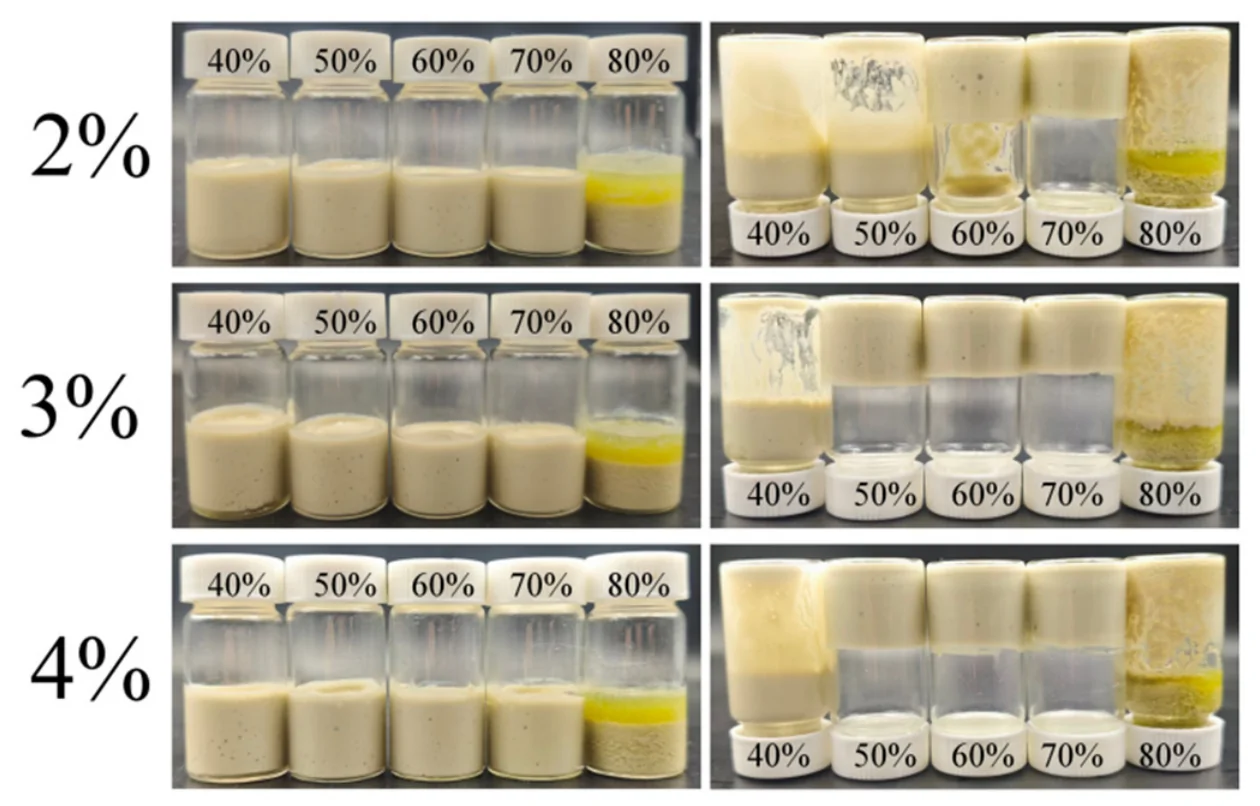
Macroscopic appearance of Pickering emulsion gels under different conditions.

The formation of Pickering emulsion gels is governed by the strong adsorption of solid particles at the oil–water interface and the establishment of particle-based networks within the continuous phase. Among the multiple parameters that regulate gelation behavior and structural stability, particle concentration and oil phase volume fraction are considered particularly decisive [26]. For this reason, we examined the macroscopic appearance of samples formulated with varying AGP concentrations and oil phase fractions, aiming to establish appropriate conditions for the formation of emulsion gels. Storage stability was monitored by visual inspection after static standing, and the self-supporting properties of the gel network were evaluated through inversion tests. As illustrated in Figure 4, structural integrity was largely preserved during storage for most of the formulations examined, while visible oil separation was observed exclusively at an oil phase fraction of 80%. This finding implies that an excessively high internal phase loading expands the total interfacial area that needs to be covered by particles; under such circumstances, the amount of AGP particles present in the system was no longer sufficient to adequately coat the surfaces of all oil droplets, thereby weakening the overall structural stability [27]. The inversion tests further revealed that the stability of the resulting emulsion gels was simultaneously governed by both AGP concentration and oil phase fraction. When AGP concentration was held constant at 2%, samples prepared with 40% and 50% oil phases displayed pronounced flow behavior upon inversion, whereas the sample containing 60% oil phase underwent only minimal displacement. This contrast indicates that an insufficient particle concentration limits the formation of a continuous and mechanically reinforced particle network within the continuous phase, thereby weakening the structural stability of the emulsion [28]. Raising AGP concentration to 3% and 4% brought about an overall improvement in structural resistance; however, partial deformation was still detected in samples containing 40% oil phase. Such deformation suggests that an oil fraction that is too low may weaken the effective interactions among droplets and consequently limit the development of a coherent network structure. In contrast, the emulsion gels containing 4% AGP exhibited excellent inversion stability within the oil phase range of 50–70%, maintaining an intact inverted structure. This behavior can be attributed to the synergistic contribution of adequate particle availability and enhanced droplet packing, which facilitated the formation of a stable three-dimensional network. However, when the oil phase fraction was further increased to 80%, all formulations lost their self-supporting ability and showed oil separation after inversion, indicating that this internal phase level exceeded the stabilization capacity of AGP particles. Based on the combined evaluation of storage stability and self-supporting performance, formulations with both structural integrity and relatively high oil-loading capacity were selected for further investigation. Specifically, the effect of AGP concentration (2–4%) was studied at a fixed oil phase fraction of 70%, while the influence of oil phase fraction (50–70%) was further evaluated in systems containing 4% AGP.

### 3.5. Droplet Size Distribution of Emulsion Gels

Droplet size distribution is closely associated with the dispersion state of oil droplets and the stability of Pickering emulsion gels, and it is commonly used to evaluate the emulsifying performance of particulate stabilizers [21]. As shown in Table 1, when the oil phase fraction (φ) was fixed at 70%, increasing the AGP concentration from 2% to 4% resulted in a continuous decrease in droplet size. Specifically, the D_50_ value decreased from 21.77 to 13.32 μm, while the D_90_ value decreased from 56.11 to 33.07 μm. The reduction in droplet size with increasing AGP concentration can be attributed to the enhanced coverage of oil droplets by AGP particles [29]. A higher particle concentration facilitates the formation of a denser interfacial layer, strengthening interfacial mechanical resistance and steric hindrance, thereby suppressing droplet coalescence and producing smaller droplets with a narrower size distribution [30]. In contrast, when the AGP concentration was maintained at 4%, increasing the oil phase fraction from 50% to 70% caused an increase in droplet size. The D_50_ increased from 8.34 to 13.32 μm, and the D_90_ increased from 18.96 to 33.07 μm. This increase was mainly associated with the enlarged total interfacial area generated by the higher oil loading. Since the amount of AGP particles remained constant, the available particles were insufficient to provide equivalent interfacial coverage for all newly formed droplets, resulting in reduced particle density at the interface. Meanwhile, the increased droplet packing under higher oil phase conditions enhanced droplet–droplet interactions and promoted localized aggregation, leading to the formation of larger droplets [31]. Overall, increasing AGP concentration contributed to droplet refinement and improved dispersion uniformity, whereas excessive oil phase loading promoted droplet enlargement. These results are consistent with previous reports on Pickering emulsions stabilized by natural plant-derived particles, in which variations in particle concentration and oil phase fraction were also found to affect droplet size, particle coverage, and emulsion stability [9,32].

### 3.6. Microstructures of Emulsion Gels

**Figure 5:**
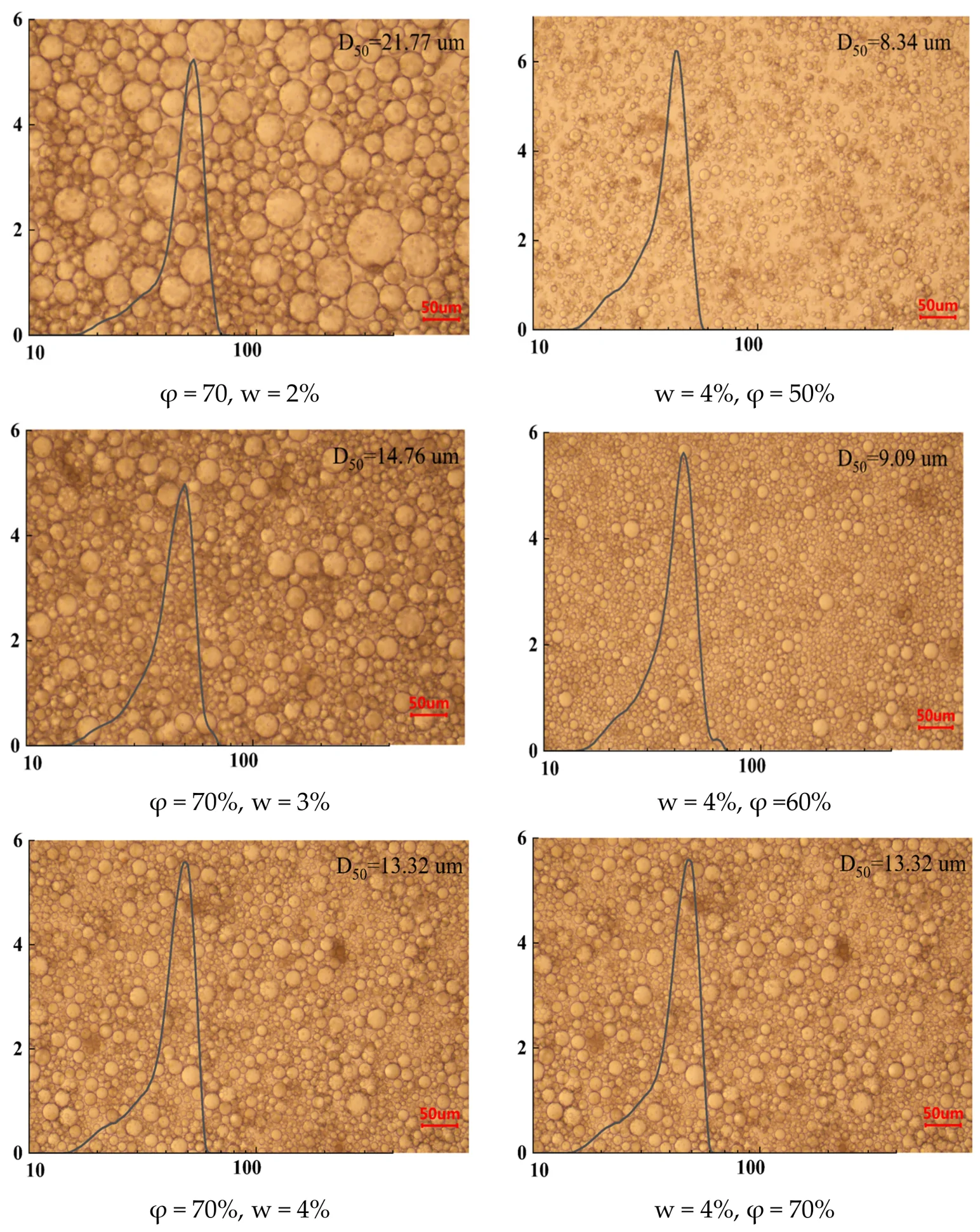
Microstructures of Pickering emulsion gels.

Optical microscopy enables direct observation of droplet shape, size distribution and spatial arrangement in Pickering emulsion gels, offering a practical means to evaluate microstructural organization and emulsification stability [33]. With the AGP concentration fixed at 4%, Figure 5 shows that raising the oil phase fraction from 50% to 60% clearly increased droplet number, while most droplets remained spherical and uniform in size. No obvious coalescence appeared, pointing to good structural integrity. When the oil fraction was further increased to 70%, larger droplets became more abundant, inter-droplet distances shrank, and occasional contact or slight aggregation was noticed. This trend suggests that a higher oil load drives tighter droplet packing and yields a more compact microstructure, in line with the droplet arrangement typical of high-internal-phase Pickering emulsions [34]. At a constant oil fraction of 70%, raising the AGP concentration from 2% to 4% progressively reduced droplet size and markedly diminished the proportion of large droplets. Meanwhile, droplet distribution became more concentrated and dispersion more uniform, resulting in a homogeneous and dense microstructure. These observations indicate that, within a suitable range, more AGP promotes effective droplet stabilization, suppresses localized aggregation and leads to a more orderly droplet arrangement, consistent with earlier work on Pickering emulsions stabilized by plant-derived particles [13]. The microscopic images agreed well with the droplet size measurements, further confirming that AGP enhanced interfacial coverage of oil droplets, whereas a higher oil phase fraction encouraged tighter droplet packing. Such structural features provide the microstructural basis for the subsequent development of stable emulsion gel networks.

### 3.7. Gel Strength of Emulsion Gels

**Figure 6:**
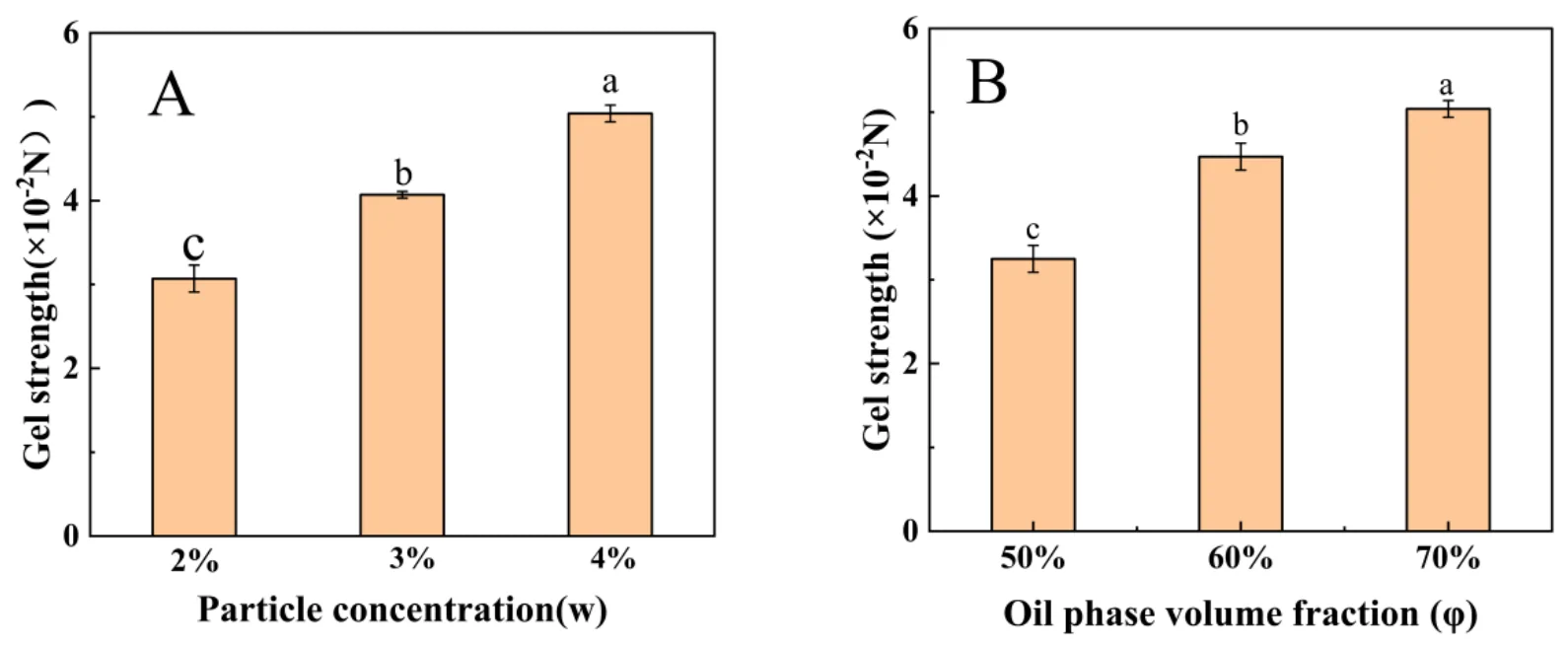
Effects of AGP concentration (**A**) and oil phase fraction (**B**) on the gel strength of Pickering emulsion gels. (**A**) The oil phase fraction was fixed at φ = 70%; (**B**) the AGP concentration was fixed at 4%. Different lowercase letters within the same figure group indicate significant differences among samples (*p* < 0.05).

Gel strength is an important parameter for evaluating the mechanical stability of Pickering emulsion gels, as it reflects the integrity of the internal three-dimensional network and the ability of the structure to withstand external deformation [35]. As shown in Figure 6A, at a fixed oil phase fraction (φ) of 70%, increasing the AGP concentration from 2% to 4% significantly enhanced the gel strength from 3.07 × 10^−2^ to 5.04 × 10^−2^ N (*p* < 0.05). The improved gel strength with increasing AGP concentration can be attributed to the increased availability of particles for adsorption at the oil–water interface, facilitating the formation of a more compact and mechanically reinforced interfacial layer. In addition, excess particles dispersed in the continuous phase may participate in interparticle interactions and bridge adjacent droplets, contributing to the development of a three-dimensional network. These combined effects enhanced the resistance of the emulsion gels against external deformation and improved their structural integrity [36]. As shown in Figure 6B, when the AGP concentration was fixed at 4%, increasing the oil phase fraction from 50% to 70% significantly increased the gel strength from 3.25 × 10^−2^ to 5.04 × 10^−2^ N (*p* < 0.05). The increased oil phase fraction resulted in a higher number of oil droplets and reduced the distance between adjacent droplets, thereby promoting the formation of densely packed droplet structures [37]. Although a higher oil loading increased the interfacial area requiring particle stabilization, the enhanced droplet packing and stronger spatial constraints among droplets contributed to the development of a stronger gel network. Consequently, the 70% oil phase system exhibited the highest gel strength, which was consistent with the compact droplet arrangement and improved structural stability observed by microscopy. Similar behavior has been reported for Pickering emulsion gels stabilized by β-glucan microgel particles, where both particle concentration and oil phase fraction jointly regulate droplet network formation and gel properties [38]. This is consistent with the present results, in which increasing AGP concentration enhanced the formation of a more compact droplet network, whereas excessive oil phase loading weakened the network structure and reduced the structural stability of the emulsion gels.

### 3.8. Microrheological Properties of Emulsion Gels

**Figure 7:**
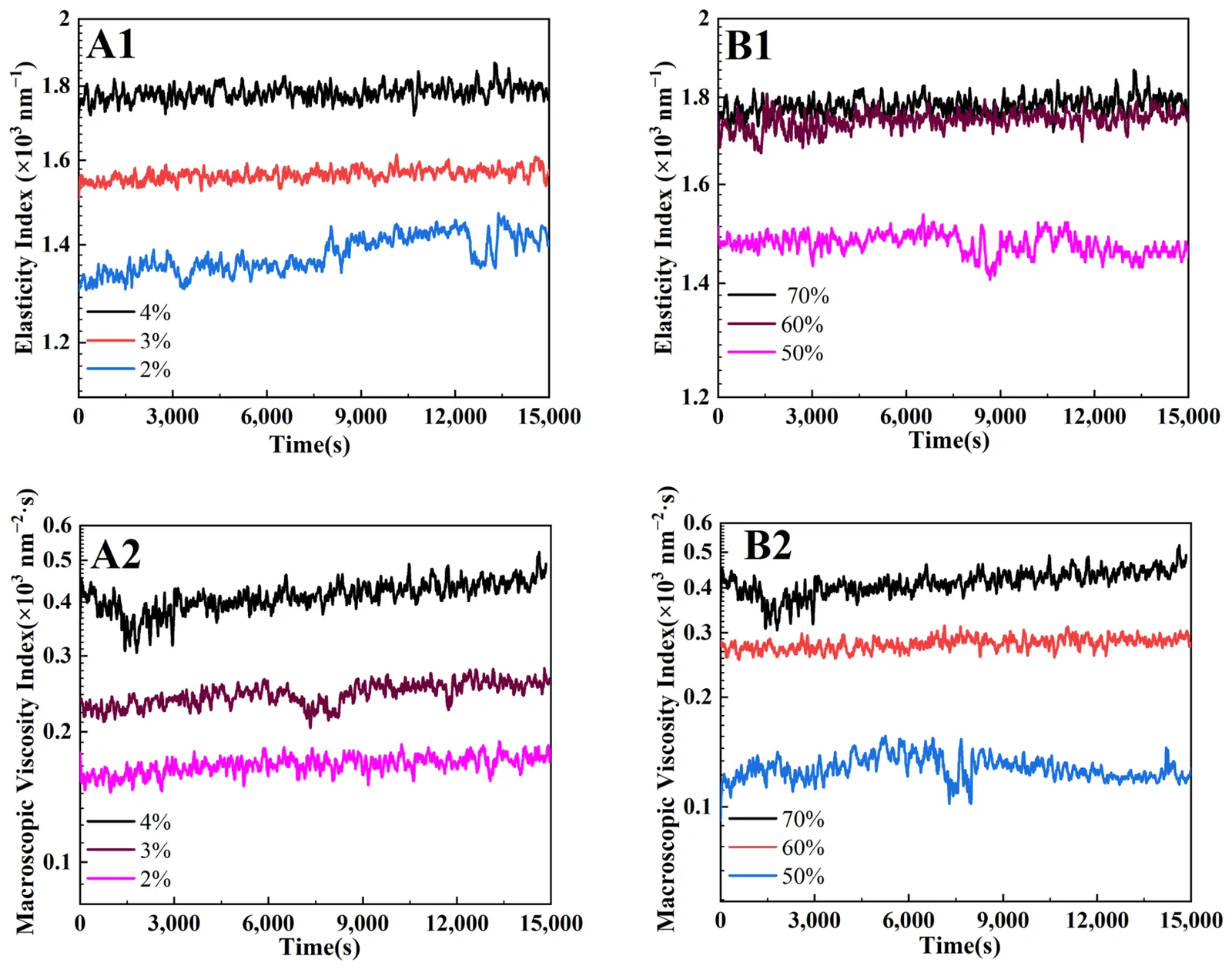
Time-dependent microrheological responses (EI and MVI) of Pickering emulsion gels at different AGP concentrations and oil phase fractions. ((**A1**,**A2**), effect of AGP concentration at φ = 70%; (**B1**,**B2**), effect of oil phase fraction at w = 4%).

Microrheological analysis enables the dynamic evaluation of the internal network stability and resistance to external disturbances of Pickering emulsion gels under nondestructive conditions [38]. The EI reflects the development of elastic structures within the emulsion gel network and its ability to recover after deformation, whereas MVI represents the degree of restriction on droplet mobility and the viscous resistance of the system. Since EI and MVI values are highly dependent on particle properties, formulation composition, dispersed phase fraction, and measurement conditions, no universal critical ranges have been established for Pickering emulsion gels. Therefore, these parameters are mainly used for comparative evaluation within the same system, and higher EI and MVI values generally reflect reduced particle mobility and enhanced structural stability [8]. As shown in Figure 7, both EI and MVI exhibited a rapid increase at the initial stage of measurement, followed by a gradual stabilization, suggesting that the emulsion gels rapidly established a relatively stable internal structure and maintained structural integrity under continuous microperturbations. As shown in Figure 7(A1,A2), when the oil phase volume fraction (φ) was fixed at 70%, increasing the AGP concentration from 2% to 4% resulted in continuous increases in both EI and MVI. This behavior suggests that higher AGP concentrations enhanced the interfacial reinforcement effect of AGP particles, resulting in a more stable emulsion gel structure and restricting droplet movement within the system [5]. As shown in Figure 7(B1,B2), increasing the oil phase fraction further enhanced both EI and MVI values. The higher internal phase content promoted closer droplet packing and increased restrictions on droplet rearrangement, thereby strengthening the ability of the gel network to withstand continuous perturbations and maintain structural stability. This observation is consistent with previous studies showing that an appropriate increase in the internal phase fraction can increase droplet packing density and contribute to the structural stability of emulsion gels [39]. The microrheological results were consistent with the gel strength measurements. The formulation containing 4% AGP exhibited both the highest gel strength and the most favorable microrheological responses, demonstrating that AGP contributes not only to the static mechanical reinforcement of Pickering emulsion gels but also to the maintenance of structural integrity under prolonged external disturbance. Although droplet jamming may contribute to the gel-like behavior of concentrated Pickering emulsions, the enhanced gel strength and microrheological responses with increasing AGP concentration indicate that AGP particles play an important role in network reinforcement.

### 3.9. Protective Effect of Emulsion Gels on Lutein Stability

**Figure 8:**
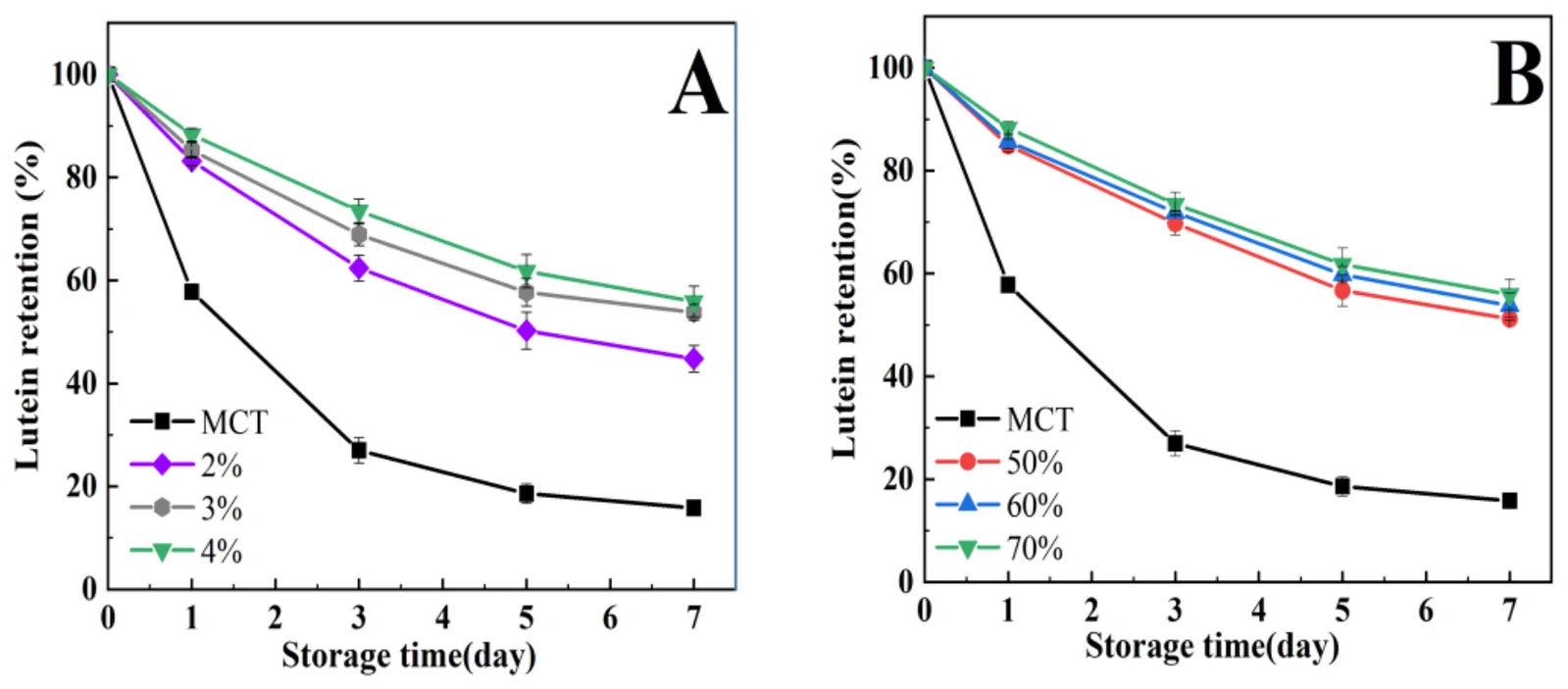
Changes in lutein retention of Pickering emulsion gels. ((**A**), Effect of AGP concentration at φ = 70%; (**B**), Effect of oil phase fraction at w = 4%).

Lutein retention is an important parameter for evaluating the protective capability of Pickering emulsion gel-based delivery systems toward lipophilic bioactive compounds. Changes in lutein retention reflect the ability of the carrier matrix to resist photooxidative degradation, reduce external exposure, and maintain the stability of encapsulated compounds [40]. As shown in Figure 8, lutein content decreased continuously in all samples during UV irradiation; however, the degradation rate varied considerably among different formulations. After 7 days of UV exposure, the MCT oil control exhibited only 14.9% lutein retention, indicating that the free oil phase provided limited protection against photooxidation and allowed rapid lutein degradation. In comparison, all AGP-containing systems showed substantially higher lutein retention under a fixed oil phase fraction of 70%. The 4% AGP system retained 55.9% of lutein after 7 days, indicating that the particle-based interfacial layer and three-dimensional gel network effectively slowed lutein loss. The flavonoids naturally present in AGP likely supplied additional antioxidant protection, creating a combined effect of physical shielding and chemical stabilization. Notably, although the 3% and 4% AGP systems reached similar retention values at day 7, the 4% formulation maintained slightly higher retention during the early irradiation period; this gap gradually narrowed with prolonged exposure. This pattern suggests that a higher AGP concentration built a more robust emulsion structure that initially limited lutein exposure to light-induced degradation [38]. Beyond a sufficient particle concentration, however, further AGP addition offered only marginal improvement in photostability. In systems prepared with 4% AGP, raising the oil phase fraction led to greater lutein retention, and the 70% oil phase system exhibited the highest stability across the irradiation period. A possible reason is that higher internal phase fractions produce a denser droplet packing structure, which restricts oxygen diffusion and suppresses light-driven oxidation, thus slowing lutein degradation [41]. Altogether, these results suggest that AGP-stabilized Pickering emulsion gels provide a protective effect against lutein degradation during UV exposure, highlighting their potential as natural delivery matrices for lipophilic bioactive compounds.

## 4. Conclusions

In this study, we prepared Pickering emulsion gels stabilized by AGP and examined how AGP modulates gel structure and protects encapsulated bioactives. AGP possessed appropriate wettability and interfacial adsorption, enabling it to form a stable particulate layer around oil droplets and providing an effective interfacial barrier for the encapsulation and protection of lipophilic bioactives such as lutein. This layer promoted homogeneous droplet dispersion and improved the structural integrity of the emulsion gel. The formulation containing 4% AGP and 70% MCT oil performed best, delivering a gel strength of 5.04 × 10^−2^ N together with enhanced microrheological stability, indicative of strong mechanical support and tolerance to persistent deformation. A higher oil fraction led to closer droplet packing and further reinforced the gel network. The AGP-stabilized emulsion gels also substantially improved the storage stability of lutein. We ascribe this protective effect to the combined action of the interfacial particle barrier and the antioxidant activity of flavonoids naturally occurring in AGP. Taken together, these findings identify AGP as a promising plant-derived particulate stabilizer that integrates structural functionality with bioactive protection, offering a green platform for delivering lipophilic bioactive compounds.

## Figures and Tables

**Table 1:** Effects of AGP concentration and oil phase fraction on the droplet size distribution of Pickering emulsion gels.

φ (%)	w (%)	D3 (μm)	D10 (μm)	D25 (μm)	D50 (μm)	D75 (μm)	D90 (μm)
70	2	1.76 ± 0.02 ^L^	4.63 ± 0.11 ^j^	11.37 ± 0.20 ^h^	21.77 ± 0.13 ^e^	36.83 ± 0.12 ^b^	56.11 ± 0.21 ^a^
	3	1.37 ± 0.09 ^L^	3.16 ± 0.03 ^k^	7.43 ± 0.48 ^i^	14.76 ± 0.42 ^f^	24.96 ± 0.44 ^d^	37.72 ± 0.71 ^b^
	4	1.26 ± 0.03 ^L^	2.93 ± 0.03 ^k^	6.95 ± 0.15 ^i^	13.32 ± 0.09 ^g^	22.48 ± 0.83 ^e^	33.07 ± 2.43 ^c^
w (%)	φ (%)	D3 (μm)	D10 (μm)	D25 (μm)	D50 (μm)	D75 (μm)	D90 (μm)
4	50	1.05 ± 0.01 ^L^	2.10 ± 0.03 ^jkl^	4.51 ± 0.04 ^i^	8.34 ± 0.07 ^g^	12.98 ± 0.10 ^f^	18.96 ± 0.84 ^d^
	60	1.14 ± 0.02 ^L^	2.35 ± 0.03 ^jk^	4.91 ± 0.07 ^i^	9.09 ± 0.17 ^g^	14.99 ± 0.25 ^e^	23.64 ± 0.44 ^b^
	70	1.26 ± 0.03 ^kL^	2.93 ± 0.03 ^j^	6.95 ± 0.15 ^h^	13.32 ± 0.09 ^f^	22.48 ± 0.83 ^c^	33.07 ± 2.43 ^a^

Values are expressed as mean ± standard deviation (*n* = 3). Different superscript letters within the same column indicate significant differences among samples (*p* < 0.05), according to one-way ANOVA followed by Duncan’s multiple range test.

## Data Availability

All the data is contained within the article, and the data presented in this study are available on request from the corresponding author.

## References

[1] Lee M.H., Kim H.D., Jang Y.J. (2024). Delivery systems designed to enhance stability and suitability of lipophilic bioactive compounds in food processing: A review. Food Chem..

[2] Cassani L., Gomez-Zavaglia A. (2024). Pickering emulsions in food and nutraceutical technology: From delivering hydrophobic compounds to cutting-edge food applications. Explor. Foods Foodomics.

[3] Funami T., Ishihara S., Maeda K., Nakauma M. (2025). Review paper: Recent development in Pickering emulsion gel technology for food and beverage applications. Food Hydrocoll..

[4] Hashemi B., Assadpour E., Wang Y., Jafari S.M. (2025). Pickering emulsion gels stabilized by protein and polysaccharide-based particles: A review of stability, synthesis, applications and prospective. Adv. Colloid Interface Sci..

[5] Gong J., Su Y., Lei J., Zhu S., He Y., Tan C.P., Liu Y., Xu Y.J. (2024). Construction and characterization of Pickering emulsion gels stabilized by *β*-glucans microgel particles. Food Hydrocoll..

[6] Xie K., He X., Chen K., Chen J., Sakao K., Hou D.X. (2019). Antioxidant properties of a traditional vine tea, *Ampelopsis grossedentata*. Antioxidants.

[7] Geng S., Jiang Z., Ma H., Pu P., Liu B., Liang G. (2021). Fabrication and characterization of novel edible Pickering emulsion gels Stabilized by dihydromyricetin. Food Chem..

[8] Song L., Zhang S., Liu B. (2022). The fabrication and characterization of Pickering emulsion gels stabilized by sorghum flour. Foods.

[9] Geng S., Wang Y., Liu B. (2024). Fabrication, Characterization and application of Pickering emulsion gels stabilized by defatted grape seed powder. Food Chem. X.

[10] Wang Y., Liu B., Ma Y., Wang C., Ma H., Geng S. (2025). Oil/water interface behavior of hesperidin methylchalcone and its application in nano-emulsions. Food Chem..

[11] Albert C., Beladjine M., Tsapis N., Fattal E., Agnely F., Huang N. (2019). Pickering emulsions: Preparation processes, key parameters governing their properties and potential for pharmaceutical applications. J. Control Release.

[12] Wang C., Wu J., Wang C., Mu C., Ngai T., Lin W. (2022). Advances in Pickering emulsions stabilized by protein particles: Toward particle fabrication, interaction and arrangement. Food Res. Int..

[13] Zhu M., Wang H., Sun T., Lv J., Wang K., Huan S., Li Z., Liu Y., Liu S., McClements D.J. (2025). Plant-based Pickering emulsions using cellulose nanofibers and soy protein isolate: Stabilization and high environmental resistance. Food Hydrocoll..

[14] Wu L., Liang Y., Lu N., Liu B. (2025). Construction, characterization, and application of immature peach powder-stabilized Pickering emulsion gel. Front. Nutr..

[15] Du L., Yue J., Zhu Y., Yin S. (2022). Production of indigo by recombinant *escherichia coli* with expression of monooxygenase, tryptophanase, and molecular chaperone. Foods.

[16] Bai L., Geng S., Zhou Y., Ma H., Liu B. (2024). Ultrasound-assisted fabrication and stability evaluation of okra seed protein stabilized nanoemulsion. Ultrason. Sonochem..

[17] Dekker R., Velandia S.F., Kibbelaar H.V.M., Morcy A., Sadtler V., Roques-Carmes T., Groenewold J., Kegel W.K., Velikov K.P., Bonn D. (2023). Is there a difference between surfactant-stabilised and Pickering emulsions?. Soft Matter.

[18] Kibbelaar H.V.M., Dekker R.I., Morcy A., Kegel W.K., Velikov K.P., Bonn D. (2022). Ethyl cellulose nanoparticles as stabilizers for Pickering emulsions. Colloids Surf. A Physicochem. Eng. Asp..

[19] Sarkar A., Dickinson E. (2020). Sustainable food-grade Pickering emulsions stabilized by plant-based particles. Curr. Opin. Colloid Interface Sci..

[20] Cui F., Zhao S., Guan X., McClements D.J., Liu X., Liu F., Ngai T. (2021). Polysaccharide-based Pickering emulsions: Formation, stabilization and applications. Food Hydrocoll..

[21] Schroën K., Shen X., Hasyyati F.I., Deshpande S., Van Der Gucht J. (2024). From theoretical aspects to practical food Pickering emulsions: Formation, stabilization, and complexities linked to the use of colloidal food particles. Adv. Colloid Interface Sci..

[22] Binks B.P., Clint J.H. (2002). Solid wettability from surface energy components: Relevance to pickering emulsions. Langmuir.

[23] Berton-Carabin C.C., Schroën K. (2015). Pickering emulsions for food applications: Background, trends, and challenges. Annu. Rev. Food Sci. Technol..

[24] Rehman A., Liang Q., Karim A., Assadpour E., Jafari S.M., Rasheed H.A., Virk M.S., Qayyum A., Rasul Suleria H.A., Ren X. (2024). Pickering high internal phase emulsions stabilized by biopolymeric particles: From production to high-performance applications. Food Hydrocoll..

[25] Zanini M., Marschelke C., Anachkov S.E., Marini E., Synytska A., Isa L. (2017). Universal emulsion stabilization from the arrested adsorption of rough particles at liquid-liquid interfaces. Nat. Commun..

[26] Geng S., Han F., Lv X., Zhang S., Ma H., Liu B. (2023). Formation mechanism of Pickering emulsion gels stabilized by proanthocyanidin particles: Experimental and molecular dynamics studies. Food Chem..

[27] Burgos-Díaz C., Garrido-Miranda K.A., Palacio D.A., Chacón-Fuentes M., Opazo-Navarrete M., Bustamante M. (2023). Food-grade oil-in-water (O/W) Pickering emulsions stabilized by agri-food byproduct particles. Colloids Interfaces.

[28] Xu W., Li Z., Li H., Sun H., Zheng S., Luo D., Li Y., Wang Y., Shah B.R. (2022). Stabilization and microstructural network of Pickering emulsion using different xanthan gum/Lysozyme nanoparticle concentrations. LWT.

[29] Chen Q.-H., Liu T.-X., Tang C.-H. (2019). Tuning the stability and microstructure of fine Pickering emulsions stabilized by cellulose Nanocrystals. Ind. Crops Prod..

[30] Benyaya M., Bolzinger M.-A., Chevalier Y., Ensenat S., Bordes C. (2023). Pickering emulsions stabilized with differently charged particles. Soft Matter.

[31] Zhao J., Guo X., Chen Z., Dai Y., Liang H., Deng Q., Li S., Zhou B. (2022). Desalted duck egg white nanogels as Pickering stabilizers for food-grade oil-in-water emulsion. Food Sci. Hum. Wellness.

[32] Sainakham M., Arunlakvilart P., Samran N., Vivattanaseth P., Preedalikit W. (2025). Formulation and stability of quercetin-loaded Pickering emulsions using chitosan/gum arabic nanoparticles for topical skincare applications. Polymers.

[33] Tenorio-Garcia E., Rappolt M., Sadeghpour A., Simone E., Sarkar A. (2024). Fabrication and stability of dual Pickering double emulsions stabilized with food-grade particles. Food Hydrocoll..

[34] Zhao G., Wang S., Yang H., Yang L., Song H., Zhang G., Liu H., He Y. (2025). Impact of oil volume fraction on stability of Pickering emulsion prepared by silica particles and soy hull polysaccharides. J. Food Eng..

[35] Fang F., Tian Z., Huang L., Cai Y., Van der Meeren P., Wang J. (2024). A Novel Pickering emulsion gels stabilized by cellulose nanofiber/dihydromyricetin composite particles: Microstructure, rheological behavior and oxidative stability. Int. J. Biol. Macromol..

[36] French D., Taylor P., Fowler J., Clegg P.S. (2015). Making and breaking bridges in a Pickering emulsion. J. Colloid Interface Sci..

[37] Li S., Zhang B., Tan C.P., Li C., Fu X., Huang Q. (2019). Octenylsuccinate quinoa starch granule-stabilized Pickering emulsion gels: Preparation, microstructure and gelling mechanism. Food Hydrocoll..

[38] Huang X., Liu B., Li Y., Huang D., Zhu S. (2024). Influence mechanism of components and characteristics on structural and oxidative stability of emulsion gel. Food Hydrocoll..

[39] Wang M., Tuo Y., Li Y., Xiao Q., Liu Y., Wu L., Zhou H., Cai Y., Zhang Y., Li X. (2025). High-pressure homogenized seaweed cellulose nanofibrils-based emulsion gel: An innovative platform for fucoxanthin encapsulation and stability improvement. Foods.

[40] Su J., Guo Q., Chen Y., Dong W., Mao L., Gao Y., Yuan F. (2020). Characterization and formation mechanism of lutein Pickering emulsion gels stabilized by β-lactoglobulin-gum arabic composite colloidal nanoparticles. Food Hydrocoll..

[41] Xu X., Li L., Ma C., Li D., Yang Y., Bian X., Fan J., Zhang N., Zuo F. (2023). Soy protein isolate-citrus pectin-gallic acid ternary composite high internal phase Pickering emulsion for delivery of β-Carotene: Physicochemical, structural and digestive properties. Food Res. Int..

